# Supplementary figures and images for: Two StAR-related lipid transfer proteins play specific roles in endocytosis, exocytosis, and motility in the parasitic protist Entamoeba histolytica
Source: PLoS Pathog. 2021 Apr 28;17(4):e1009551. doi: 10.1371/journal.ppat.1009551 (PMC8109825; doi:10.1371/journal.ppat.1009551)

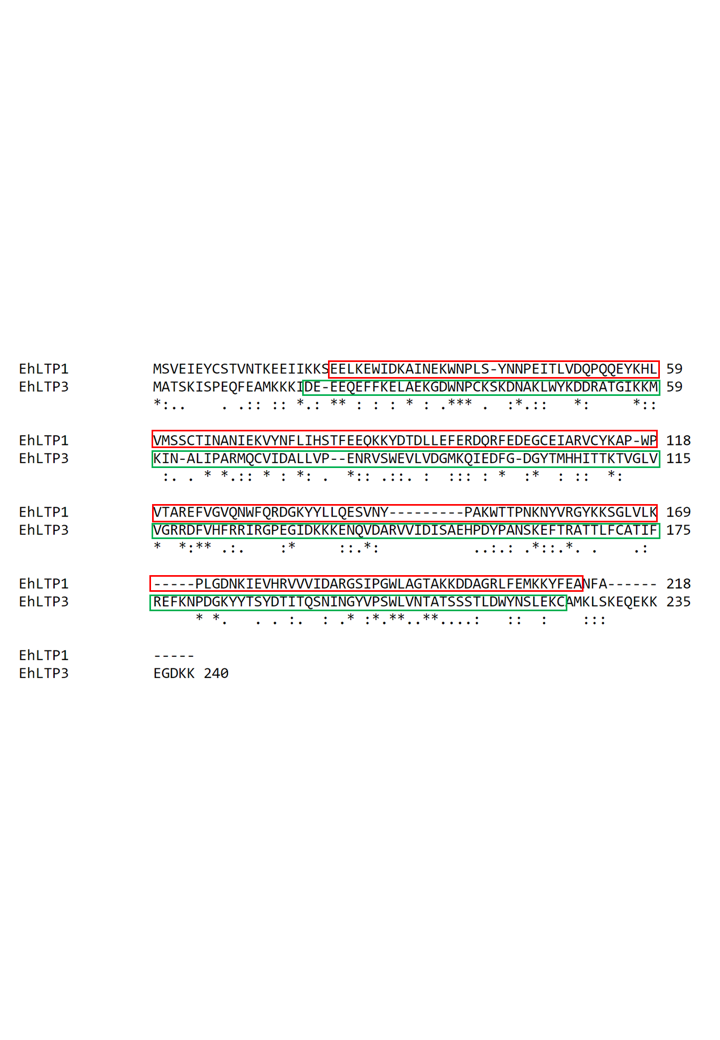

Supplement: S1 Fig — Red and Green boxes indicate the “STARD” domain of EhLTP1 and EhLTP3, respectively. (TIFF) [file ppat.1009551.s001.tiff]

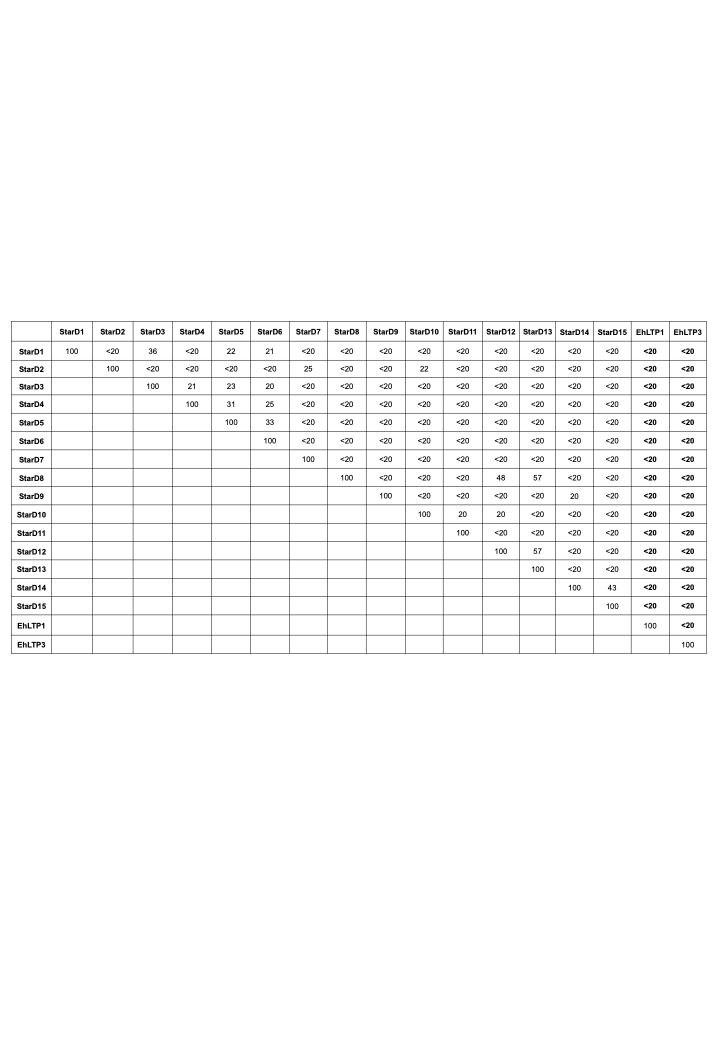

Supplement: S2 Fig — Identities below 20 percent are shown as ‘<20’. Note that both EhLTP1 and EhLTP3 are highly diverse from their counterparts from humans (identities were below 20 percent). (TIFF) [file ppat.1009551.s002.tiff]

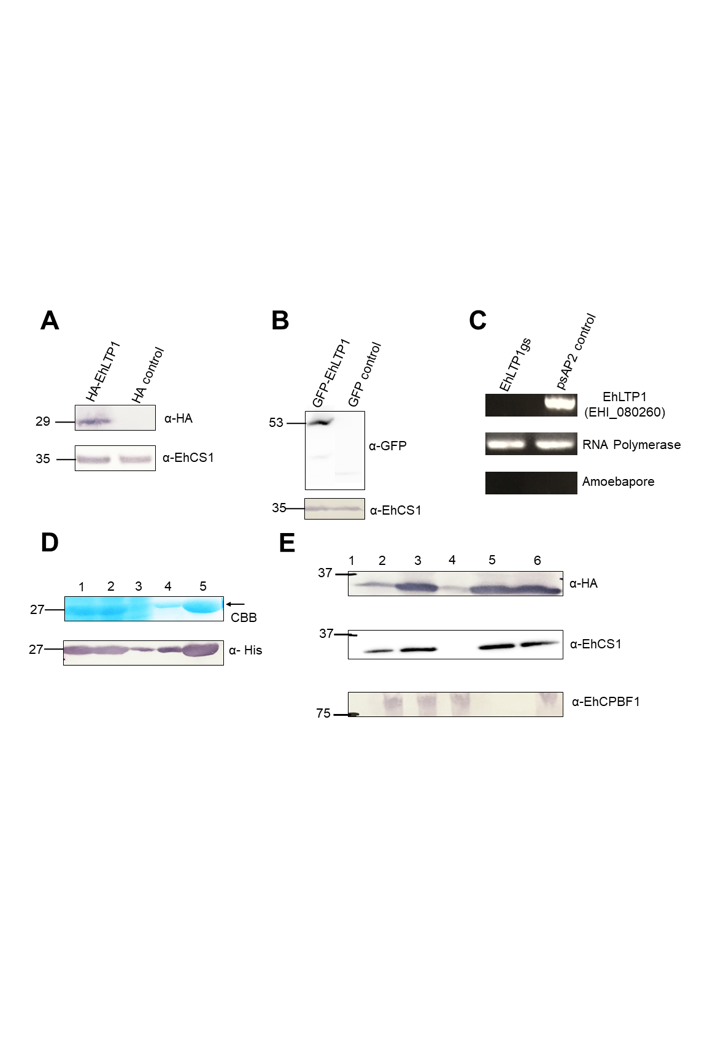

Supplement: S3 Fig — (A) Expression of HA-tagged full length EhLTP1 (HA-EhLTP1) in E. histolytica trophozoites detected by anti-HA antibody. (B) Expression of GFP-tagged full length EhLTP1 (GFP-EhLTP1) in E. histolytica trophozoites detected by anti-GFP antibody. (C) The expression of EhLTP1 was silenced by antisense small RNA-mediated transcriptional gene silencing in G3 strain of E. histolytica. The expression was monitored by reverse transcriptase PCR. Lane 1, EhLTP1gs; Lane 2 vector control line (psAP2-Gunma). The RNA polymerase II gene was used as loading control. The silencing of amoebapore gene in transfected G3 strain was also checked. (D) His tag recombinant EhLTP1 (rEhLTP1) was expressed and purified. Expression of recombinant protein (rEhLTP1) was verified by CBB and also by immunoblot analysis with anti-His antibody. Lane 5, rEhLTP1 (approximately 27kD) indicated by an arrow. (E) Sub-cellular fractionation of transformant expressing HA-EhLTP1 followed by immunoblot analysis. Sub-cellular fractionation was performed as described in Methods section. Immunoblot analysis was performed using anti-HA mouse monoclonal antibody (1:1000). Anti-CPBF1 (cysteine protease binding family protein 1) (1:500) and anti-CS1 (cysteine synthase 1) (1:500) rabbit antisera were used as organelle membrane and cytosolic markers, respectively. Lane 1: marker, lane 2: 5000×g pellet fraction (p5), lane 3: 5000×g supernatant fraction (s5), lane 4: 100,000×g pellet fraction (p100), lane 5: 100,000×g supernatant fraction (s100) and lane 6: total lysate (TL). (TIFF) [file ppat.1009551.s003.tiff]

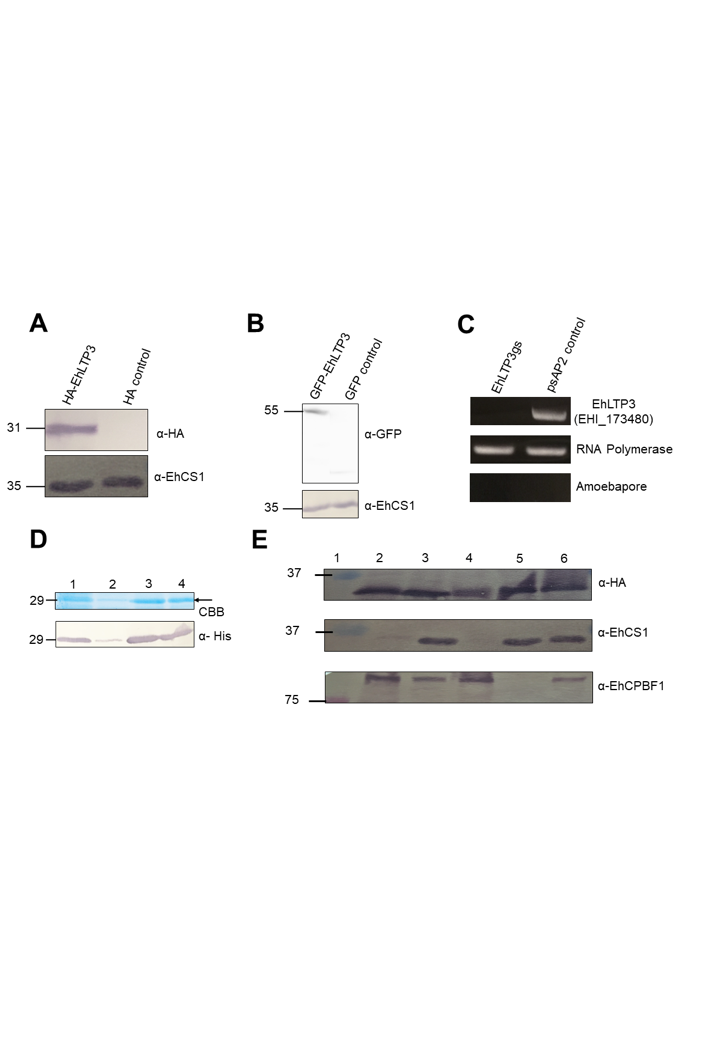

Supplement: S4 Fig — (A) Expression of HA-tagged full length EhLTP3 (HA-EhLTP3) in E. histolytica trophozoites detected by anti-HA antibody. (B) Expression of GFP-tagged full length EhLTP3 (GFP-EhLTP3) in E. histolytica trophozoites detected by anti-GFP antibody. (C) The expression of EhLTP3 was silenced by antisense small RNA-mediated transcriptional gene silencing in G3 strain of E. histolytica. The expression was monitored by reverse transcriptase PCR. Lane 1, EhLTP3gs; lane 2, vector control line (psAP2-Gunma). The RNA polymerase II gene was used as loading control. The silencing of amoebapore gene in transfected G3 strain was also verified. (D) His tag recombinant EhLTP3 (rEhLTP3) was expressed and purified. Expression of recombinant protein (rEhLTP3) was verified by CBB and also by immunoblot analysis with anti-His antibody. Lane 4, rEhLTP3 (approximately 29 kD), indicated by an arrow. (E) Sub-cellular fractionation of transformant expressing HA-EhLTP3 followed by immunoblot analysis. Sub-cellular fractionation was performed as described in Methods section. Immunoblot analysis was performed using anti-HA mouse monoclonal antibody (1:1000). Anti-CPBF1 (cysteine protease binding family protein 1) (1:500) and anti-CS1 (cysteine synthase 1) (1:500) rabbit antisera were used as organelle membrane and cytosolic markers, respectively. Lane 1: marker, lane 2: 5000×g pellet fraction (p5), lane 3: 5000×g supernatant fraction (s5), lane 4: 100,000×g pellet fraction (p100), lane 5: 100,000×g supernatant fraction (s100) and lane 6: total lysate (TL). (TIFF) [file ppat.1009551.s004.tiff]

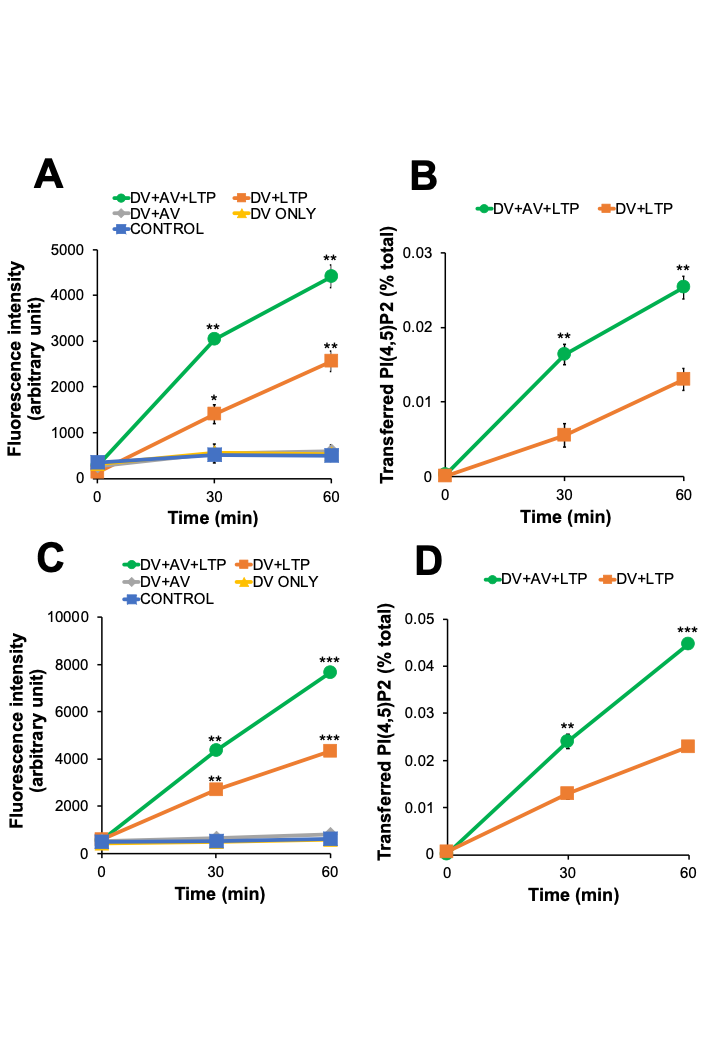

Supplement: S5 Fig — In vitro transfer of PtdIns(4,5)P2 from donor to acceptor vesicles in the presence of rEhLTP1 (A, B) and rEhLTP3 (C, D). Donor vesicles (DV) (1.5 μl) containing PtdIns(4,5)P2 were incubated in separate reaction without acceptor vesicles (AV) and recombinant proteins (“DV ONLY”), with 0.5 μg of either rEhLTP1 or rEhLTP3 (“DV+LTP”), with 3 μl of AV (“DV+AV”), or with 3 μl of AV and 0.5 μg of either rEhLTP1 or rEhLTP3 (“DV+AV+LTP”) at room temperature. Fluorescence units were measured after 30 and 60 min of incubation. The percentage of transfer of PtdIns(4,5)P2 (B, D) were calculated from (A, C), respectively as described in Methods. The experiments were conducted in duplicates three times (n = 3 with error bars indicating standard deviations). Statistical comparisons were made by Student’s t-test (*P ≤ 0.05, **P ≤ 0.005, ***P ≤ 0.0005). The y axis represents arbitrary fluorescence units. (TIFF) [file ppat.1009551.s005.tiff]

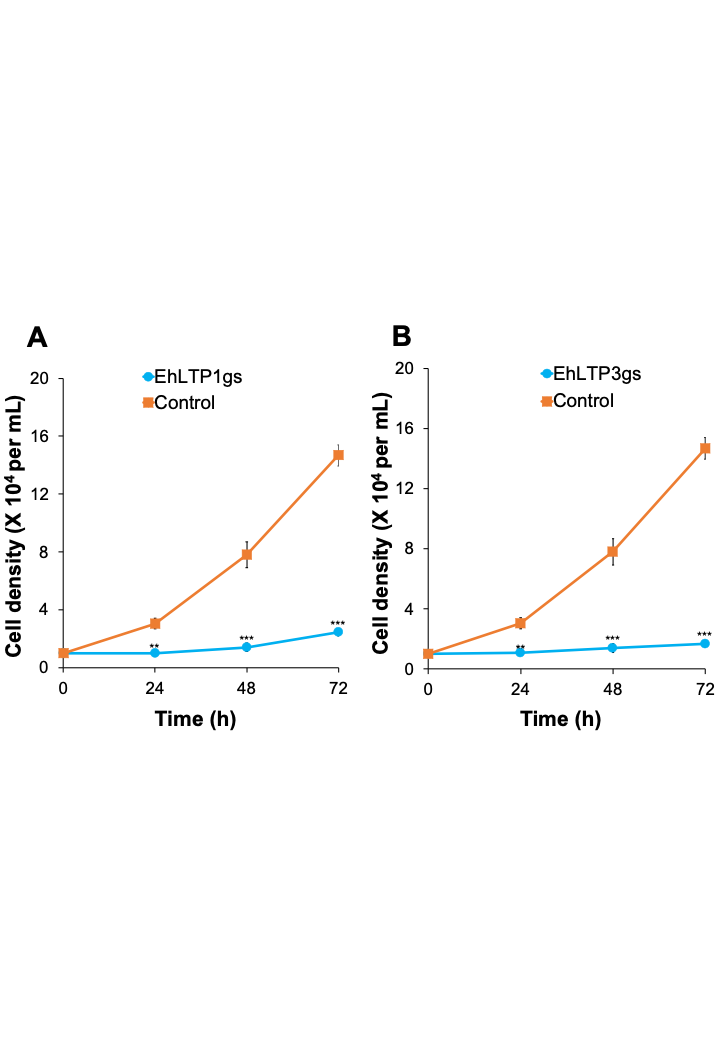

Supplement: S6 Fig — Growth of the E. histolytica strains in which EhLTP1 (A) or EhLTP3 gene (B) was silenced. Gene kinetics of EhLTP1gs (A), EhLTP3gs (B), and their mock transformant (with psAP2 control) during 72 h cultivation in BI-S-33 medium is shown. The experiment was repeated three times independently in duplicates (N = 3 with error bars indicating standard deviations). Statistical comparisons were made by Student’s t-test (*P ≤ 0.05, **P ≤ 0.005, ***P ≤ 0.0005). (TIFF) [file ppat.1009551.s006.tiff]

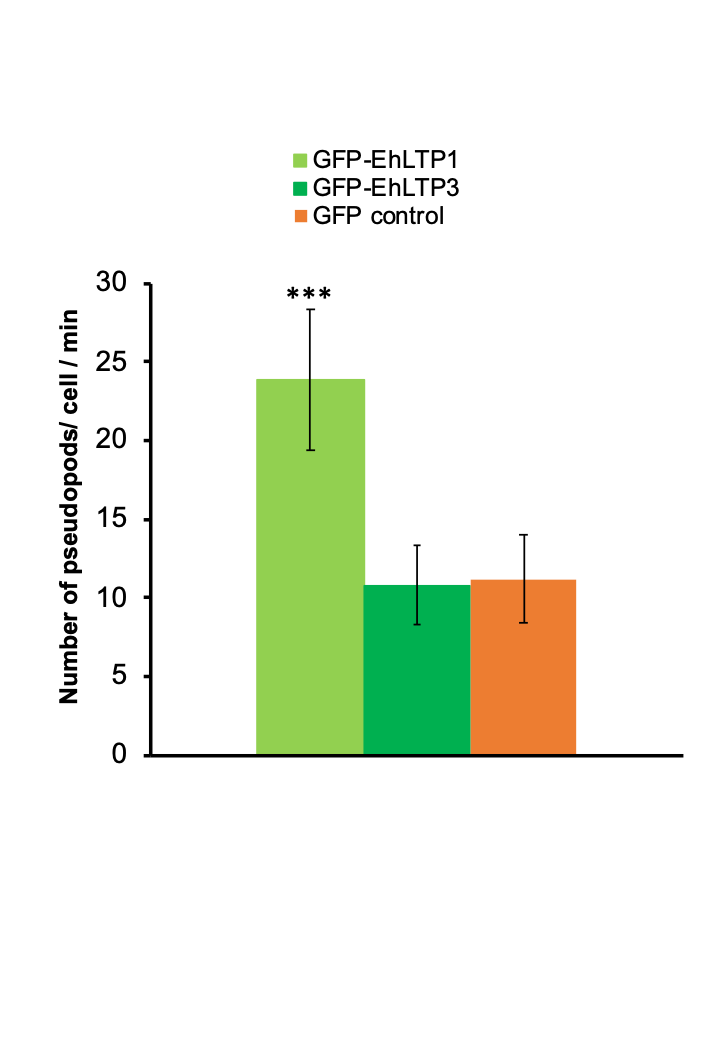

Supplement: S7 Fig — The number of newly forming pseudopods were quantified in E. histolytica overexpressing transformants (GFP-EhLTP1 or GFP-EhLTP3) and their corresponding vector control (GFP control) cell lines in a specified time. The results were expressed as average number of pseudopods per cell per min. The experiment was repeated three times independently in duplicates (N = 3 with error bars indicating standard deviations). Statistical comparisons were made by Student’s t-test (*P ≤ 0.05, **P ≤ 0.005, ***P ≤ 0.0005). (TIFF) [file ppat.1009551.s007.tiff]

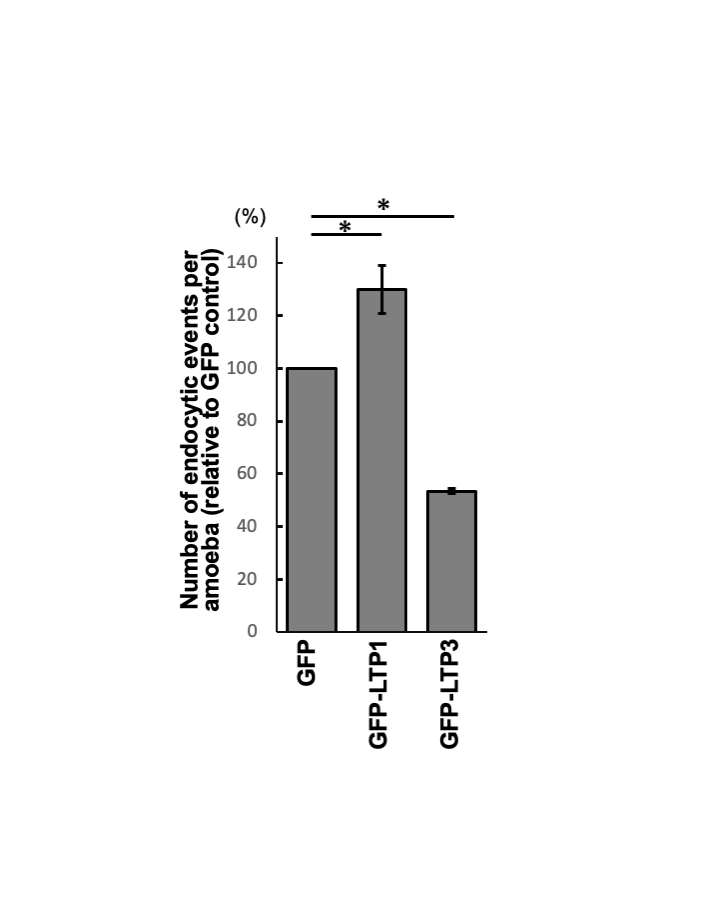

Supplement: S8 Fig — A total of ten to fifteen GFP-positive cells of each transformants were randomly selected each in three independent experiments incubated with BI-S-33 medium containing 2 μg/ml RITC-dextran. The time-lapse images were captured at 2.0 s intervals for 6.5 mins. The number of invagination was counted and expressed as average number of invaginations per cell per minute in percentage relative to that in GFP control. The experiment was repeated three times independently (N = 3 with error bars indicating standard deviation). Statistical comparisons were made by Tukey test (*P ≤ 0.02). (TIFF) [file ppat.1009551.s008.tiff]

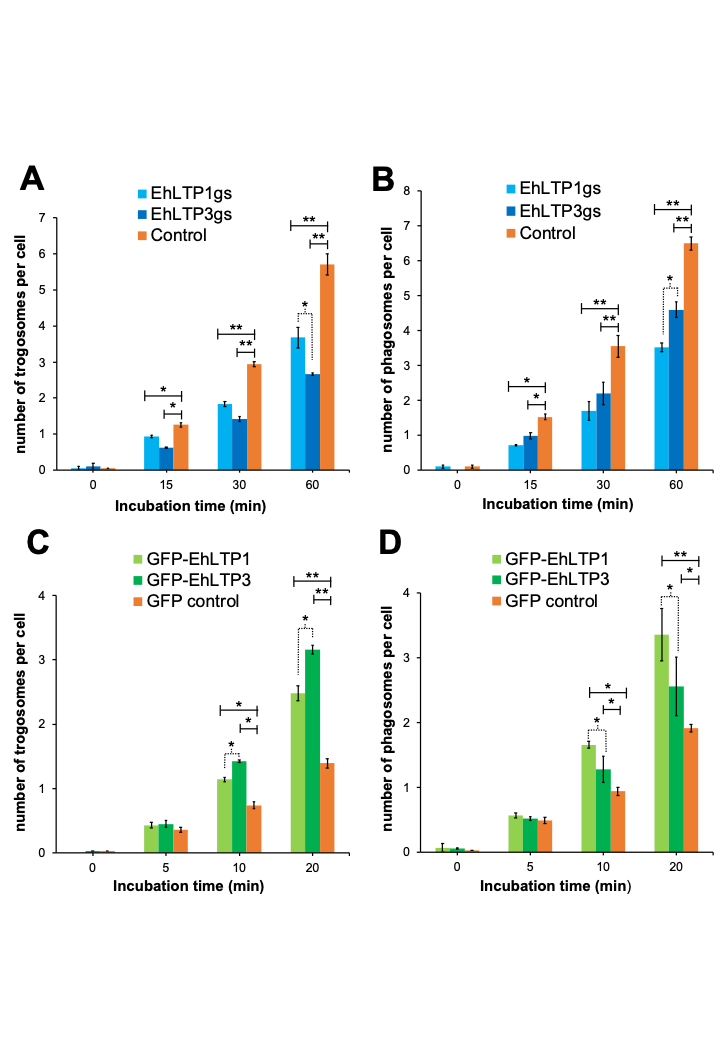

Supplement: S9 Fig — Effects of gene silencing (A, B) and overexpression (C, D) of EhLTP1 and EhLTP3 on trogocytosis (A, C) and phagocytosis (B, D) efficiency of E. histolytica trophozoites. Cell Tracker Orange labelled CHO cells were co-incubated with E. histolytica transformants for indicated time as described in Methods. A total of thirty-five cells from each microscopic field (a total of seven microscopic field) of each transformants were randomly selected in each independent experiment and the total number of trogosomes or phagosomes in those cells were counted at indicated time points. The results were expressed as average number of trogosomes or phagosomes per cell. The experiment was repeated three times independently in duplicates (N = 3 with error bars indicating standard deviation). Statistical comparisons were made by Student’s t-test (*P ≤ 0.05, **P ≤ 0.005, ***P ≤ 0.0005). (TIFF) [file ppat.1009551.s009.tiff]

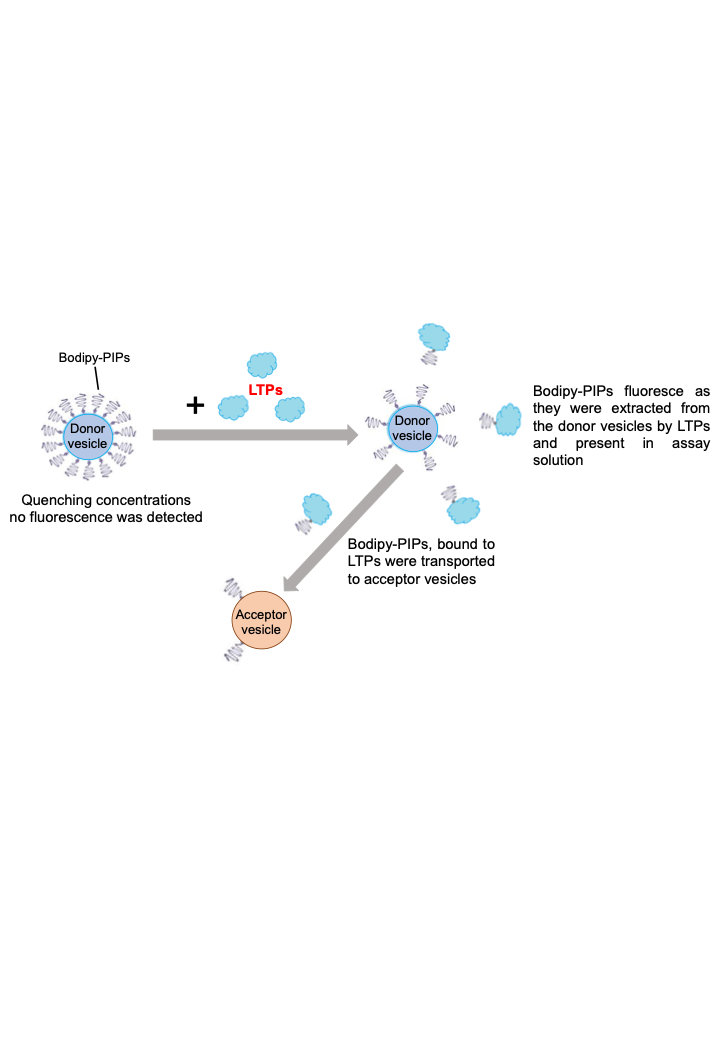

Supplement: S10 Fig — (TIFF) [file ppat.1009551.s010.tiff]

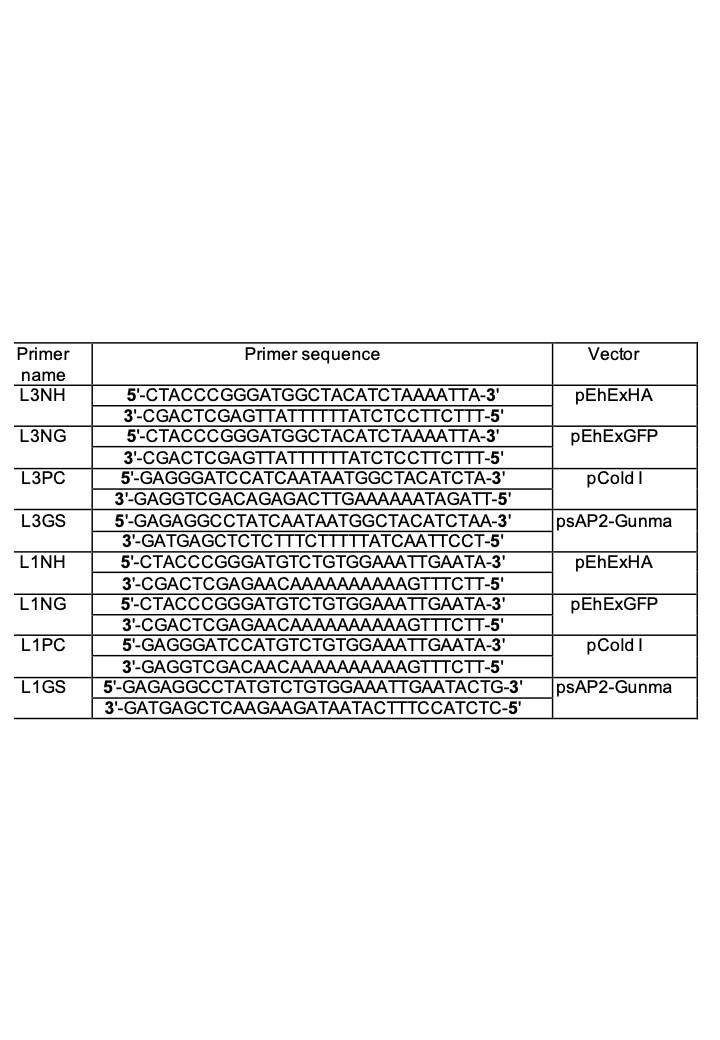

Supplement: S1 Table — (TIFF) [file ppat.1009551.s011.tiff]
