# Supplementary material for: Two StAR-related lipid transfer proteins play specific roles in endocytosis, exocytosis, and motility in the parasitic protist Entamoeba histolytica
Source: PLoS Pathog. 2021 Apr 28;17(4):e1009551. doi: 10.1371/journal.ppat.1009551 (PMC8109825; doi:10.1371/journal.ppat.1009551)
Supplement: Attachment — Submitted filename: Das_Rebuttal.docx [file ppat.1009551.s021.docx]

We appreciate both referees for all constructive comments and Associate Editor for his/her understanding of our situation that does not allow us to carry out some of suggested experiments (also please see below).

In revision, we made the following major changes:

1. We carried out quantitation of the frequency of fluid-phase endocytosis and added the data as new S8 Fig, and modified the text accordingly. (see below comment #4 of referee #1). To complete this part, one author has been added.

2. We renumbered some of supplementary figures as previous S7 Fig was misplaced (it should have been placed after S4 Fig in the order of appearance). We renumber it to S5 Fig, and changes supplementary figure numbers (S7→S5; S5→S6; S6→S7; a new S8 inserted; S8→S9; S9→S10; S10→S11). 3. The new supplementary figure S8, as stated above, to show the number of invaginations for fluid-phase endocytosis was added.

4. The discussion section was largely reduced according to the suggestion.

Regarding point #2 above, although we understand and appreciate the major criticism by Reviewer #1, we did not perform the suggested experiment. Reviewer #1 raised his/her concern for off-target effect caused by overexpression and gene silencing of EhLTP1 and 3, and requested additional experiments: RNA seq analysis of both overexpressing and gene-silenced strains as well as their respective control lines. We are reluctant to carry out this suggested experiment. The reasons are as follow:

1) We validated the phentypes using both overexpressing and gene-silenced transformant lines, which showed apparently reversed, reasonable phenotypes (i.e., upregulation vs downregulation), as anticipated, for the majority of the examined phenotypes. Thus, it is VERY UNLIKELY that the observed phenotypes are due to off-target effects on irrelevant genes. It is unlikely that off-target effects were observed for the identical gene or a group of similar genes in the transformants that had been created by different genetic mechanisms, i.e., episomal plasmid-based overexpression vs. antisense small RNA-mediated transcriptional gene silencing).

2) Transcriptome study by RNA seq analysis is being planned as a downstream study following this study, because such approach may give information on genetic interaction between LTPs and PI-metabolizing enzymes such as PI kinases and PI phosphatases that LTPs may present the bound lipids to, and PI effectors downstream such as cytoskeletal and membrane traffic machinery. Thus, if allowed, we would like to include this part of study in the subsequent study in future.

3) Due to Covid-19 endemics, our laboratory resources have been largely diminished, including funds that is needed for RNA seq analysis (six samples x replicates).

We consulted the Associate Editor about the issue prior to submission of the revised manuscript through PLoS Pathogens Editorial Office, and received the confirmation that we do not need to address this specific criticism for resubmission.

Below please see below our point-to-point response to each comment.

Comment #1:

**Major Issues: Key Experiments Required for Acceptance**

Reviewer #1: This is a very cleanly conducted study that will be of interest to a wide readership. I do have one comment, however. There is, at least theoretically, the possibility that the production of transfectants leads to off target effects. Thus, there is a slight possibility that the determined partly different activities of both molecules are due to the corresponding off target effects. Therefore, it is important to determine the expression profile of the silencer and overexpression transfectants using next generation sequencing (RNAseq).

**Our response**: We fully understand the concern and appreciate the suggestion. However, we did not perform this experiment. Please see above our explanation.

Comment #2

Principal weaknesses

1. The steroidogenic acute regulatory (STAR) protein is present in phospholipid transfer proteins and plays a major role in the supply of cholesterol to mitochondria. LTP1 and LTP3 in E. histolytica (an organism lacking mitochondria) contain the STAR domain and appear to be deleted from other lipid binding domains. Unfortunately, the presentation of LTP1 and LTP3 in this article is very poor. In the absence of amoeba STAR structural data, which exists from other organisms, there are many approaches to research the characteristics of STAR and other domains in these candidate proteins in E. histolytica. In addition, the authors do not discuss the absence of signal for cholesterol (or ceramide very abundant in the amoeba) following their lipid overlay test (figure 1) and also the fact that LTP1 binds to cardiolipin is not discussed.

**Our response**: Structural analysis of EhLTPs is beyond the scope of this study and will be performed in future. We have added comments on the lack of binding of EhLTPs to either cholesterol or ceramide and the unexpected binding of EhLTP1 to cardiolipin as follows:

" A interaction of rEhLTP1 with cardiolipin and sulphatides was also observed (Fig 1a), which may be potentially due to their negative charge." (correction underlined) (Page 5, line 145)

"Neither rEhLTP1 nor rEhLTP3 bound to cholesterol, which is consistent with the premise that oxysterol-binding protein-related proteins [22] are potentially involved in cholesterol transport." (Page 5, lines 148)

Comment #3:

2. In the liposome experiment (which was the only mechanistic investigation of LTPs in this work), LTP1 and LTP3 have activity to extract PI (4) P and PtdIns (4,5) P2 from donor liposomes and transfer them to acceptor liposomes. The authors stated that LTP3 prefers PtdIn (4,5) P2 over PI (4) P, while LTP3 has a weak but reversed preference for PI (lane 169). For this reader, the data in the figures 2 A and B are quite similar to C and D since PtdIns (4,5) P2 as a donor is not specified.

**Our response**: For clarification, lipid extraction and transfer activity toward PtdIns (4) P or PtdIns (4,5) P2 are shown in Fig 2 or previous S7 Fig (new S5 Fig in revision), respectively. Please note the Y axis in both figures. For instance, EhLTP1 shows: "DV+AV+LTP" PI(4)P (green) at 60 min reads about 5,000 (arbitrary unit) (Fig 2A) while "DV+AV+LTP" PI(4,5)P2 reads about 4,000 (new S5 Fig, A). In contrast, EhLTP3 shows: "DV+AV+LTP" PI(4,5)P2 (green) at 60 min reads about 2,000 (arbitrary unit) (Fig 2C) while "DV+AV+LTP" PI(4,5)P2 reads about 8,000 (new S5 Fig, C). Thus, what we claim for isotype-dependent preference of EhLTP1 and EhLTP3 toward PI(4)P or PI(4,5)P2 was correct.

We apologize our mistake in the last sentence (Line 165). For clarity, We rewrote the sentence as follow:

"These data provide direct biochemical evidence that EhLTP1 and 3 have activity to extract PI(4)P and PtdIns(4,5)P_2_ from donor liposomes and transfer to acceptor liposomes; moreover, EhLTP3 has clear preference to PtdIns(4,5)P_2_ over PI(4)P, while EhLTP1 has almost comparable activity towards PI(4)P and PtdIns(4,5)P_2_ " (correction shown underlined) (Page 6, line 174)

Comments #4:

3. In line 313, the authors argue that the overexpression of LTP3 causes an increase in invagination of the plasma membrane. Looking carefully at the videos this phenotype is not obvious. In video 1 (LTP1), I notice invaginations in images 340, 435, 483. The number of invaginations per cell must be counted in an equivalent period of time.

**Our response**: We appreciate this valuable suggestion. We have added quantitative data of invagination of GFP-EhLTP1-, GFP-EhLTP3-overexpressing and control strains (new S8 Fig.). We also added the description of the results (Lines 324-):

"To see if increased endocytosis of RITC-dextran (see above) and augmented membrane invagination caused by EhLTP3 overexpression is attributable to increase in the number of endocytic events, we measured the invagination events per cell per min (S8 Fig.). Expression of GFP-EhLTP3 caused 46.4±0.9% reduction (mean±standard deviation, n=3) in the number of invagination events compared to that in GFP control. In contrast, expression of GFP-EhLTP1 caused 30.2±9.1% increase in the number of invagination events compared to that in GFP control. These data, together with the quantitative data of endocytosed RITC-dextran, are consistent with the premise that GFP-EhLTP3 overexpression causes bigger and less frequent bites, whereas GFP-EhLTP1 overexpression causes smaller and slightly more frequent bites. Therefore, both EhLTP1 and EhLTP3 are involved in endocytosis of the fluid-phase marker, but in reciprocal manners. Overall, EhLTP3, and to a lesser extent, EhLTP1, are involved in fluid-phase endocytosis (Fig 3)."

The fact that GFP-EhLTP3 overexpression caused bigger bites but reduced invagination events, may be counter-intuitive, but probably explains how the movies (S1, S4, and S8 movies) gave a biased impression.

We also changed the heading of the paragraph to: "Overexpression of EhLTP3 increases in the volume of endocytosis of the fluid-phase maker, but represses the number of invagination events." (Line 319)

Comments #5:

4. The characteristics described in the text (line 352) are not observed in the video microscopy 7. These data need precision. Pynosomes are visible but not colocalization with RITC dextran particles. Control using either the LTP1 construct and / or WT cells may help to narrow the observation

**Our response**: After further careful reexamination of the movies including the one provided as S7 Movie, we agree to the referee's point that the characteristics described in this paragraph including the subheading are not well observed in the video microscopy 7. We have removed the description of specific recruitment of EhLTP3 to "apical portion" of the pinosome. Furthermore, we also examined localization of GFP-EhLTP1 during dextran pinocytosis. Membrane association of GFP-EhLTP1 was not observed and the movie was added as a new movie (new S8 Movie). We accordingly modified the main text as follow:

"EhLTP3 is recruited to the pinocytic site on the plasma membrane and the pinocytic cup during pinocytosis. Since our endocytosis assay (see above) indicated that both EhLTP3 and EhLTP1 are reversely involved in pinocytosis (endocytosis of fluid-phase markers) (Fig 3), we further investigated on the cellular localization of EhLTP3 during pinocytosis of RITC dextran in live imaging. GFP-EhLTP3 was recruited to the pinocytic site of a plasma membrane invagination during pinocytosis, and associates with the pinocytic cup till its closure (S7 Movie). In contrast, this recruitment to the pinocytic site was not observed for GFP-EhLTP1 (S8 Movie). These data further supported our premise that EhLTP3 is involved primary in endocytosis of fluid phase markers." (page 12, lines 366-)

Accordingly, previous S8 Movie (GFP-expressing transformant) has been renamed as S9 Movie.

Comment #6:

5. The localization dynamics of PIs in E. histolytica during trogo- and phagocytosis has been extensively studied through their binding effectors, including several proteins studied by the author’s team. There is a long speculation in the discussion on this point; however, there is no information on the potential interaction of LTP with these effectors either by colocalization experiments (using their commercially available constructs and antibodies identifying PIs) and / or in their discussion.

**Our response**: We appreciate the valuable suggestions. Particularly, we believe that investigation on the enzymes that utilizes PI4P and PI(4,5)P2, such as PI 3-kinases and the ones that produce them such as PTEN is important and currently undergoing. However, we believe inclusion of analysis of such effectors is beyond the scope of current study. We will describe role of other essential PI effectors in future.

Comment #7:

**Part III – Minor Issues: Editorial and Data Presentation Modifications**

Reviewer #1: The number of E. histolytica infections and deaths has increased dramatically in recent years due to improved hygienic conditions. Unfortunately, there are only limited recent figures for this. (GBD 2013 Mortality and Causes of Death Collaborators, Global, regional, and national age-sex specific all-cause and cause-specific mortality for 240 causes of death, 1990-2013: a systematic analysis for the Global Burden of Disease Study 2013, Lancet 2015

**Our response**: We have included the suggested reference in Introduction.

Comment #8:

Reviewer #2: Concerning the manuscript body

-The material and methods section must follow the same organization (order) as the results section

**Our response**: We have changed the order of subsections of Materials and Methods according to the order of subsections in Results.

Comment #9:

- From line 86. There is a confusing notion which, in my opinion, equates all phenomena based on ingestion of something with endocytosis; but endocytosis is a well-defined mechanistic cellular process. I think trogocytosis is a different process from endocytosis and that they cannot be included as an example of endocytosis, it's confusing. Even phagocytosis, which in ancient literature appears to be an endocytic process, is now well defined because mechanically speaking, it does not follow the endocytic pathways in the early stages. Moreover, no one knows if the pieces of human cells taken by the amoeba are ingested by endocytosis. I recommend to read these two recent articles PMID: 30504135 to PMID: 32366574 which clearly allows to conclude in distinction of these concepts. Even reference 43 in your text makes this difference. Please edit the text along the manuscript with this distinction in mind.

**Our response**: We appreciate the suggestions. We have modified the text throughout the manuscript accordingly particularly with two points in mind:

1. when internalization of a cell (or a portion of the cell) is described, we avoided to use the term "endocytosis", and referred to "phagocytosis", "trogocytosis", or "trogo-/phagocytosis" (e.g., lines 85, 106). When internalization of fluid-phase markers or extracellular ligands recognized by surface receptor(s) is described, the term of "endocytosis" is used (e.g., line 105). When endocytosis of the fluid-phase marker is involved, we occasionally used the term "pinocytosis". Its explanation was given in the first version of our manuscript: "Pinocytosis (endocytosis as measured by incorporation of fluid-phase markers) is a process used for the uptake of nutrients from the extracellular environment [21]". (line 195)

We appreciate the suggestions for two references (PMID: 30504135 and PMID: 32366574), which were carefully read in order to make clearer definition of the terms in this manuscript. We also added the two indicated references in the main text, as they provide clear definitions of endocytic pathways, in the sentence that reads:

"Although endocytosis and phagocytosis are well defined at the molecular level in model organisms [32,33], the regulation of trogocytosis is poorly understood [24,34]*.*" (line 291).

We also made the following corrections:

Line 28: "endocytic processes including" was removed.

Line 191: "the fluid-phase marker" was added for clarification.

Line 414, "endocytic processes such as trogo- and phago-....": "endocytic processes such as" has been removed.

Line 509: " that regulates endocytosis, phago-/trogocytosis, and ": the word underlined inserted.

Comment #10

-Line 100. Strain c6 is not a reference strain. Please correct.

**Our response**: We have deleted "reference".

Comment #11:

-The discussion is too long, dispersed and should gain in interest if a concordance between the literature and the results can be established (see comment 3). The diagram shown in Figure S10 might help the main text. You may want to consider placing it in the main article.

**Our response**: We made S10 Fig as new figure (new Fig. 8 in revision). We largely rewrote the first four paragraphs of Discussion (lines 382-474 of the original version) to streamline Discussion according to the suggestion. The word counts of this part (lines 401-432 of the revised version) was extensively reduced (1110 to 396 words). We tried to establish concordance between the experimental data and the literature throughout the text.

Comment #12:-The assessment of line 536 "may also provide a potential target for the development of new therapies against eukaryotic pathogens" is greatly overstated as it is not clear how new therapies against amoebiasis might include LTPs.

Our response: We have softened our argument. (line 511)
